# From mechanisms to future therapy: a synopsis of isolated REM sleep behavior disorder as early synuclein-related disease

**DOI:** 10.1186/s13024-025-00809-0

**Published:** 2025-02-11

**Authors:** Ambra Stefani, Elena Antelmi, Dario Arnaldi, Isabelle Arnulf, Emmanuel During, Birgit Högl, Michele M. T. Hu, Alex Iranzo, Russell Luke, John Peever, Ronald B. Postuma, Aleksandar Videnovic, Ziv Gan-Or

**Affiliations:** 1 Medical University Innsbruck, Innsbruck, Austria; 2 DIMI Department of Engineering and Medicine of Innovation, University of Verona, Verona, Italy; 3 Clinical Neurophysiology, IRCCS Ospedale Policlinico San Martino, Genoa, Italy; 4 DINOGMI, University of Genoa, Genoa, Italy; 5 Sleep Clinic, Pitié-Salpêtrière Hospital, APHP - Sorbonne University, Paris, France; 6 Paris Brain Institute, Paris, France; 7 Department of Neurology, Icahn School of Medicine at Mount Sinai, New York, NY USA; 8 Division of Neurology, Nuffield Department of Clinical Neurosciences, Oxford University, Oxford, UK; 9 Sleep Unit, Neurology Service, Hospital Clínic de Barcelona, IDIBAPS, CIBERNED: CB06/05/0018-ISCIII, Universitat de Barcelona,, Barcelona, Spain; 10 Department of Cell and System Biology, University of Toronto, Toronto, ON Canada; 11 Department of Neurology and Neurosurgery, McGill University, Montreal, QC Canada; 12 The Neuro (Montreal Neurological Institute-Hospital), Montreal, QC Canada; 13 Department of Neurology, Massachusetts General Hospital, Harvard Medical School, Boston, USA; 14 Department of Human Genetics, McGill University, Montreal, QC Canada

## Abstract

Parkinson disease (PD), dementia with Lewy bodies (DLB) and multiple system atrophy are synucleinopathies, characterized by neuronal loss, gliosis and the abnormal deposition of α-synuclein in vulnerable areas of the nervous system. Neurodegeneration begins however several years before clinical onset of motor, cognitive or autonomic symptoms. The isolated form of REM sleep behavior disorder (RBD), a parasomnia with dream enactment behaviors and excessive muscle activity during REM sleep, is an early stage synucleinopathy. The neurophysiological hallmark of RBD is REM sleep without atonia (RWSA), i.e. the loss of physiological muscle atonia during REM sleep. RBD pathophysiology is not fully clarified yet, but clinical and basic science suggest that ɑ-syn pathology begins in the lower brainstem where REM atonia circuits are located, including the sublaterodorsal tegmental/subcoeruleus nucleus and the ventral medulla, then propagates rostrally to brain regions such as the substantia nigra, limbic system, cortex. Genetically, there is only a partial overlap between RBD, PD and DLB, and individuals with iRBD may represent a specific subpopulation. A genome-wide association study identified five loci, which all seem to revolve around the *GBA1* pathway. iRBD patients often show subtle motor, cognitive, autonomic and/or sensory signs, neuroimaging alterations as well as biofluid and tissue markers of neurodegeneration (in particular pathologic α-synuclein aggregates), which can be useful for risk stratification. Patients with iRBD represent thus the ideal population for neuroprotective/neuromodulating trials. This review provides insights into these aspects, highlighting and substantiating the central role of iRBD in treatment development strategies for synucleinopathies.

## Introduction

REM sleep behavior disorder (RBD) is a parasomnia characterized by dream enactment in the setting of loss of physiological muscle atonia during REM sleep, with vocal and/or motor behaviors that may lead to injuries during sleep period [1]. RBD is diagnosed more commonly in males [2]; older studies reported a male to female ratio of 9:1, while more recent studies demonstrate this ratio to be closer to 2:1 [3–5] Estimated prevalence based on recent population-based studies is around 1% among individuals over 50 years of age [2, 6, 7].

Diagnostic criteria proposed by the American Academy of Sleep Medicine (AASM) require repeated episodes of vocalization or complex motor behaviors during REM sleep and the demonstration of REM sleep without atonia (RSWA). RSWA is the neurophysiological signature of RBD, documented from a video polysomnography. The AASM and the International RBD Study Group have established scoring guidelines for RSWA [8]. Several RBD questionnaires have been developed and might be useful for screening purposes [9] but their specificity is low [10]. Different conditions may mimic RBD, the most common being non-REM parasomnias, sleep-related hypermotor epilepsy, periodic limb movement disorder [11], severe obstructive sleep apnea., sleep terrors in slow wave sleep [12], benzodiazepine-induced automatism and amnesia, and nocturnal dissociative (psychogenic) behaviors.

RBD can be classified into isolated (formerly idiopathic) RBD (iRBD) and secondary RBD. Isolated RBD occurs in the absence of an associated clinically manifest neurological disease. Secondary RBD occurs in the presence of associated disorders such as neurodegenerative disorders (in particular synucleinopathies), autoimmune disorders, narcolepsy, focal brainstem lesions, and other comorbidities. A high proportion of individuals affected by iRBD eventually develop synuclein-specific neurodegenerative disorders (i.e., synucleinopathies) such as PD, DLB or MSA [13]. The largest international study to date that examined 1280 patients with RBD and revealed a phenoconversion rate of 6.3% per year; 73% of this study cohort developed a synucleinopathy within a 12-year follow up period [14]. These findings are in line with several longitudinal single-center studies that examined the evolution of iRBD [15, 16].

This review aims at providing an overview of RBD with a focus on its isolated form as early synucleinopathy, highlighting how RBD research can shed light into synucleinopathy pathophysiology and provide insights into neuromodulating or neuroprotective treatments for these neurodegenerative diseases from the earliest stages.

### Clinical features of RBD

During sleep, patients with RBD act out violent dreams. They may box, kick and shout, usually in the second part of the night (when REM sleep predominates). The eyes are closed and patients generally stay in bed, but sometimes sit or jump out of bed. Unlike sleepwalkers, they do not get up and walk around. When they wake up, they are usually not confused and often remember a dream that matches their actions [17]. In the dream scenario, patients are often being attacked by humans or animals and fight back, protect their family, argue or play sport [18]. Sometimes they laugh, speak, or make non-violent gestures [19]. The movements are fast, less coordinated than waking movements, and often jerky. Of note, movements can be rapid and vigorous even in PD patients who are severely bradykinetic during wakefulness [20]. The frequency of behaviors varies considerably from night to night. Many are not noticed by patients and their spouses. Due to the violent nature of these behaviors, patients may injure themselves or their loved ones, leading to abrasions and fractures [21].

During the daytime, there are no immediate consequences of nocturnal behaviors. However, patients with iRBD may have mild motor and non-motor symptoms [22, 23]. This shows that neurodegeneration also affects pathways not involved in sleep. In the motor domain`, signs of parkinsonism may include minimal cogwheel rigidity when the examiner moves the wrist`, mild hypokinesia or bradykinesia`, loss of arm swing when walking`, and subtly decreased face and vocal expression [24]. Independently from the presence of cognitive complaints, a comprehensive neurocognitive battery in iRBD patients often demonstrate reduced attention and executive functions, and later memory and visuospatial decline, starting even before the development of mild cognitive impairment and several years before dementia diagnosis in those patients who progress to DLB [25, 26]. In the sensory domain, patients frequently present with hyposmia, altered taste perception, and color vision deficits [22, 27]. Common autonomic problems include blood pressure drop when standing, constipation, urinary problems, and erectile dysfunction [22]. In the psychiatric domain, anxiety, low mood, and apathy can also be observed [28]. The olfactory and autonomic signs often appear well before motor and cognitive signs in the progression from iRBD to overt parkinsonism/dementia [23, 29].

People with PD who also have RBD most commonly have the rigid-akinetic form of parkinsonism, more non-motor signs and hallucinations [30], and progress more quickly to PD dementia [31], motor disability [32], and falls [33], suggesting a more severe form of PD.

### Neurophysiology of RBD

Neurophysiology provides classical and quantifiable biomarkers of RBD and neurodegeneration in patients with isolated or no longer isolated RBD [13]. Video-polysomnography (V-PSG) records multiple neurophysiological biomarkers in various channels, including electroencephalography (EEG), electromyography (EMG) and electrooculography (EOG). These have been used to better characterize RBD and predict conversion [34] and also proven useful in prodromal RBD [35]. V-PSG is not only the required diagnostic instrument for RBD (demonstrating RSWA is mandatory for the diagnosis), but capable to provide a unique set of multiple specific biomarkers in RBD. Its full potential has not yet been fully exploited. Both the AASM and the IRBDSG recommend RSWA quantification in the chin (tonic and phasic EMG activity [36, 37], attributed to different brain circuits [13]) and upper extremities. As early as in their first descriptions of RBD Schenck and Mahowald highlighted the need to record upper extremities EMG [38], as confirmed by later research [37, 39].

As RSWA quantification requires skilfulness and time, automatic methods have been developed [8]. The Innsbruck Group proposed a simple method with recording of flexor digitorum superficialis surface EMG only, validated as sensitive and specific to detect RBD in patients with an AHI < 15/h [40]. EEG is another key neurophysiological signal, and researchers have early appreciated waking and sleep EEG slowing to be a marker of ongoing neurodegeneration in RBD [41–43]. More recent studies investigated REM sleep EEG microstructural alterations [44], hypnodensity, and increased phase synchronization in RBD [45].

Rapid eye movements are another neurophysiological hallmark occurring in clusters during REM sleep. In RBD, violent and elaborate movements occur mainly during REM sleep with REMs, compared to background jerking [46]. REM density was first investigated by Lapierre and Montplaisir [36], and later work showed that patients with iRBD have less slow and rapid eye movements during wake after sleep onset on nocturnal polysomnography [47]. These neurophysiological signals are useful markers of disease progression, instruments to define or even predict specific phenotypes of neurodegeneration, to predict timing of conversion and potential marker of treatment response. The full potential of neurophysiology might go far beyond the description of events and details. Well-done state-of-the-art neurophysiology recordings of EMG, EEG and EOG in RBD is able to provide most highly useful and precise information, which can be used to monitor progress quantitatively, quantify and characterize treatment response, characterize different subtypes (e.g. with or without cognitive impairment, with our without psychiatric/hallucinatory symptoms).

Furthermore, neurophysiology is a very promising instrument to detect the often very subtle onset of RBD in its prodromal stages very sensitively and precisely [13], in particular using automated analysis as mentioned above. Other neurophysiological aspects in RBD (e.g. blink reflex variants, intracortical facilitation to gain insight into brain circuit function or periodic leg movements) have been summarized elsewhere [48–50].

### Isolated RBD as an early stage of synucleinopahty

Parkinson disease (PD), dementia with Lewy bodies (DLB) and multiple system atrophy (MSA) are neurodegenerative diseases characterized by neuronal loss, gliosis and the abnormal deposition of the protein α-synuclein (α-syn) in surviving cells of multiple vulnerable areas of the nervous system. In PD and in DLB, these deposits are found as Lewy bodies and Lewy neurites in neurons, whereas in MSA they are found as cytoplasmic inclusions in glial cells [51, 52]. In PD, it is hypothesized that α-syn pathology begins when its misfolded form migrates from the olfactory bulb and/or gut to the brain where it spreads in a cell-to-cell fashion across the brainstem, subcortical areas and the neocortex. This may lead to progressive neurodegeneration and the sequential appearance of hyposmia, dysautonomia, RBD, parkinsonism, hallucinations and dementia [53]. Although α-syn pathology is a central element in synucleinopathies, other factors are involved including mitochondrial dysfunction, lysosomal dysfunction, oxidative stress and inflammation [54].The etiology of synucleinopathies is complex wherein individual, genetic and environmental factors are involved [51, 52].

PD is the most common synucleinopathy and occurs in 1–2% of the population over 60 years of age. The diagnosis of PD is made when parkinsonism (bradykinesia plus rigidity and/or resting tremor) is noticeable, corresponding to 60–70% neuronal loss in the substantia nigra and 80% dopaminergic deficit in the striatum [55]. DLB is the second most common dementia after Alzheimer’s disease and accounts for about 5–15% of dementia cases. Additional features in DLB are parkinsonism, fluctuations, and visual hallucinations [56]. PD and DLB are overlapping conditions characterized by different clinical starting points, with dementia preceding parkinsonism in DLB, and more than 80% PD cases developing dementia (at least on year after parkinsonism onset) when considering 20-year follow-up time [57]. Neuropathology of DLB and PD dementia are similar, consisting of widespread Lewy pathology in the brainstem, limbic system and neocortex plus coexisting Alzheimer’s disease pathology and multiple age-related pathologies [58]. MSA is a less frequent condition with a more aggressive course characterized by dysautonomia in combination with parkinsonism and/or cerebellar syndrome [59].

The synucleinopathies have a prodromal period of many years where the neuropathological process (e.g., deposits of mis-folded α-syn), has started and where some symptoms occur before the onset of the cardinal symptoms which define these diseases (i.e., dementia, parkinsonism and dysautonomia) [60–62]. Among these symptoms is RBD, that occurs as a prodromal feature in 30–50% of PD [63], 50% of DLB and 50% of MSA patients [3, 64, 65].

### Disease mechanisms in RBD and insights into synucleinopathies – evidence from basic science

**Fig. 1:**
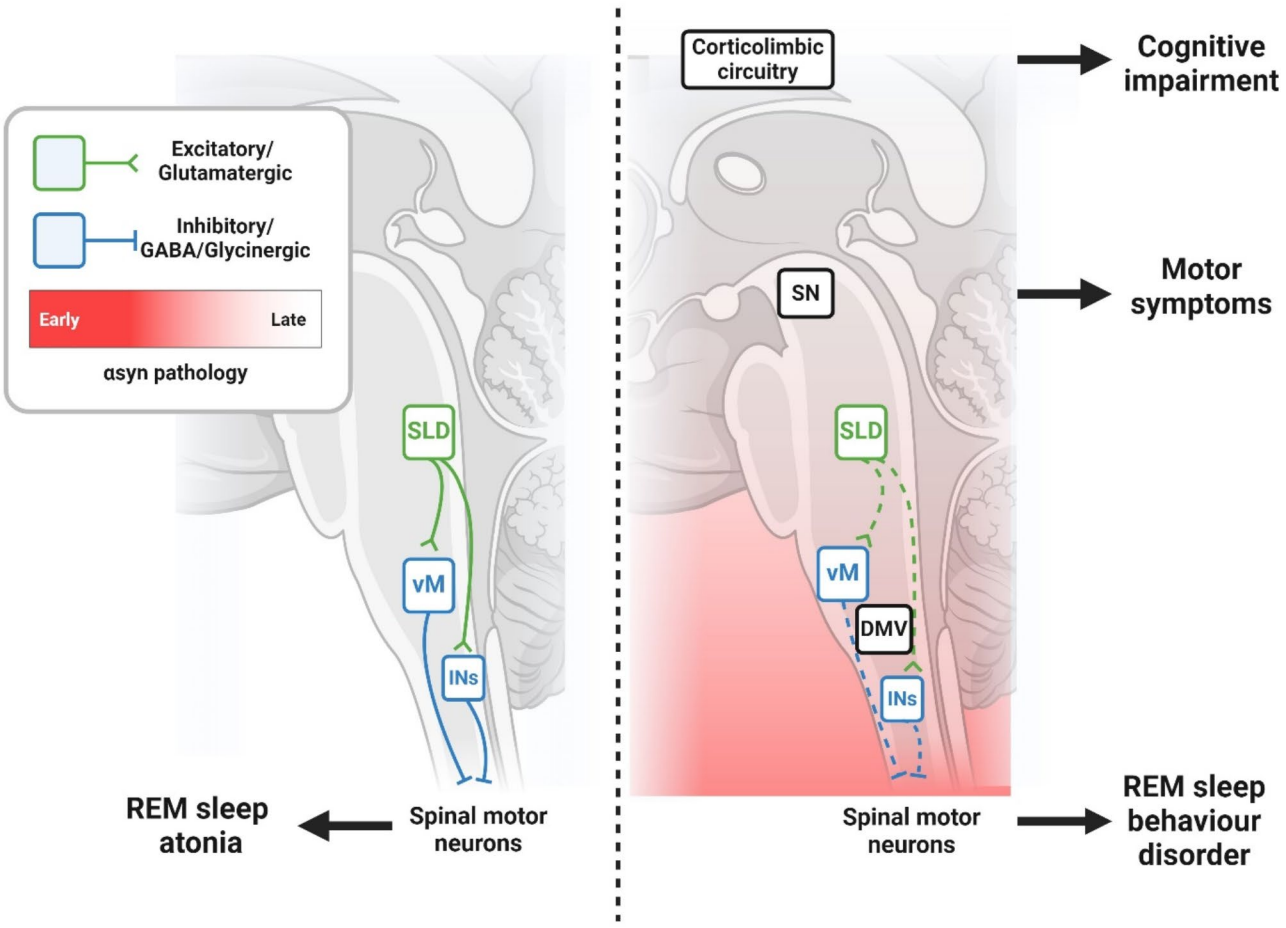
Synucleinopathic degeneration of REM sleep circuits are hypothesized to underlie RBD. Alpha-synuclein aggregation begins in the caudal brainstem and propagates from cell to cell in a caudal-rostral fashion. At prodromal stages of disease, pathology initially develops within the brainstem substrates for REM atonia, including the sublaterodorsal tegmental (SLD) neurons and ventral medulla (vM) neurons. During healthy REM sleep, SLD cells excite vM cells that inhibit spinal motoneurons to induce motor atonia. In RBD, α-syn-mediated dysfunction of these cells leads to loss of REM sleep atonia and the motor behaviors of RBD. Eventually, pathology spreads rostrally towards regions classically associated with the synucleinopathies such as the substantia nigra pars compacta (SNpc) and corticolimbic circuits, where it causes the cardinal motor and cognitive manifestations of these disorders. Figure created with BioRender.com

Despite its clinical importance, our understanding of the mechanisms underlying RBD remains incomplete. Given that loss of REM sleep atonia defines RBD, the brainstem circuits responsible for REM sleep atonia are likely implicated in its pathophysiology. The core of these circuits includes the sublaterodorsal tegmental nucleus (SLD; called the subcoeruleus nucleus in humans) and the gigantocellular and magnocellular nuclei in the ventral medulla (vM, Fig. 1).

Experimental work in rats demonstrates that glutamatergic neurons in the SLD become selectively active during REM sleep [66, 67]. Recent data indicate that these glutamate-releasing neurons express corticotropin-releasing hormone-binding protein (Crhbp) [68]. These cells project to and excite GABA/glycine neurons in the vM [69–71] which, in turn, results in the release of GABA and glycine onto somatic motoneurons in the spinal cord and cranial nerve nuclei in the brainstem, causing muscle atonia [72–74]. Disrupting SLD or vM neurons through lesions and genetic inactivation results in the loss of REM atonia and motor behaviors reminiscent of human RBD [66, 75–80]. In humans, ischemic or inflammatory lesions involving structures homologous to the SLD and vM also lead to RBD [81–84]. Thus, converging lines of evidence suggest that REM sleep atonia is generated by the same brainstem circuits in animals and humans, and that dysfunction of these structures could underlie RBD symptomatology. Of note, impairment of the afferents of the key brainstem nuclei that regulate REM sleep atonia (e.g. amygdala in anti-leucine-rich glioma-inactivated 1 encephalitis, and lateral posterior hypothalamus in narcolepsy) may also trigger RBD in humans with an intact brainstem [85]. 

While the exact cause of dysfunction in REM atonia-regulating neurons is unclear, the notion that RBD is a prodromal stage of synucleinopathies implies synucleinopathic degeneration. Correspondingly, post-mortem examination of four iRBD patient brains revealed α-syn inclusions and neuronal loss in areas corresponding to the SLD and vM [86–88] whereas imaging data indicates cell loss in these regions [89, 90]. Furthermore, recent post-mortem evidence reveals a loss of Crhbp-expressing neurons, but not cholinergic neurons in the SLD of PD patients with RBD, thus implicating specific SLD cell types in RBD pathophysiology [68]. These data support the hypothesis that αsyn-mediated degeneration of REM atonia circuits causes RBD. Neuropathological evidence shows that isolated RBD patients exhibit a brainstem-predominant pathological distribution [86, 87], which contrasts with the widespread pathology seen in advanced disease [91]. This suggests that the spread of ɑ-syn pathology from the caudal brainstem could drive clinical progression from prodromal (i.e. iRBD) to advanced stages of the synucleinopathies (Fig. 1).

Recent research has focused on whether experimentally induced αsyn pathology can trigger RBD symptoms in animal models. In mice, the transgenic overexpression of αsyn increases muscle activity during REM sleep and elicits an RBD-like phenotype [92], supporting the link between α-syn pathology and RBD. Additionally, chemogenetic activation of Crhbp-expressing neurons in the SLD was shown to alleviate early reductions in NREM and REM sleep amounts seen in these transgenic mice [68]. However, the effects of this intervention on RBD-like behaviors that typically emerge at later stages in this model, were not assessed in the study. Another study found that direct inoculation of the SLD with α-syn fibrils induced αsyn pathology in SLD neurons, which triggered RBD-like behaviors in mice [93]. This same study also showed that the spread of ɑ-syn pathology to the substantia nigra led to parkinsonism. These findings strongly support the hypothesis that iRBD is caused by ɑ-syn pathology in the REM atonia circuit and establish a mechanistic link between iRBD and PD.

**Fig. 2:**
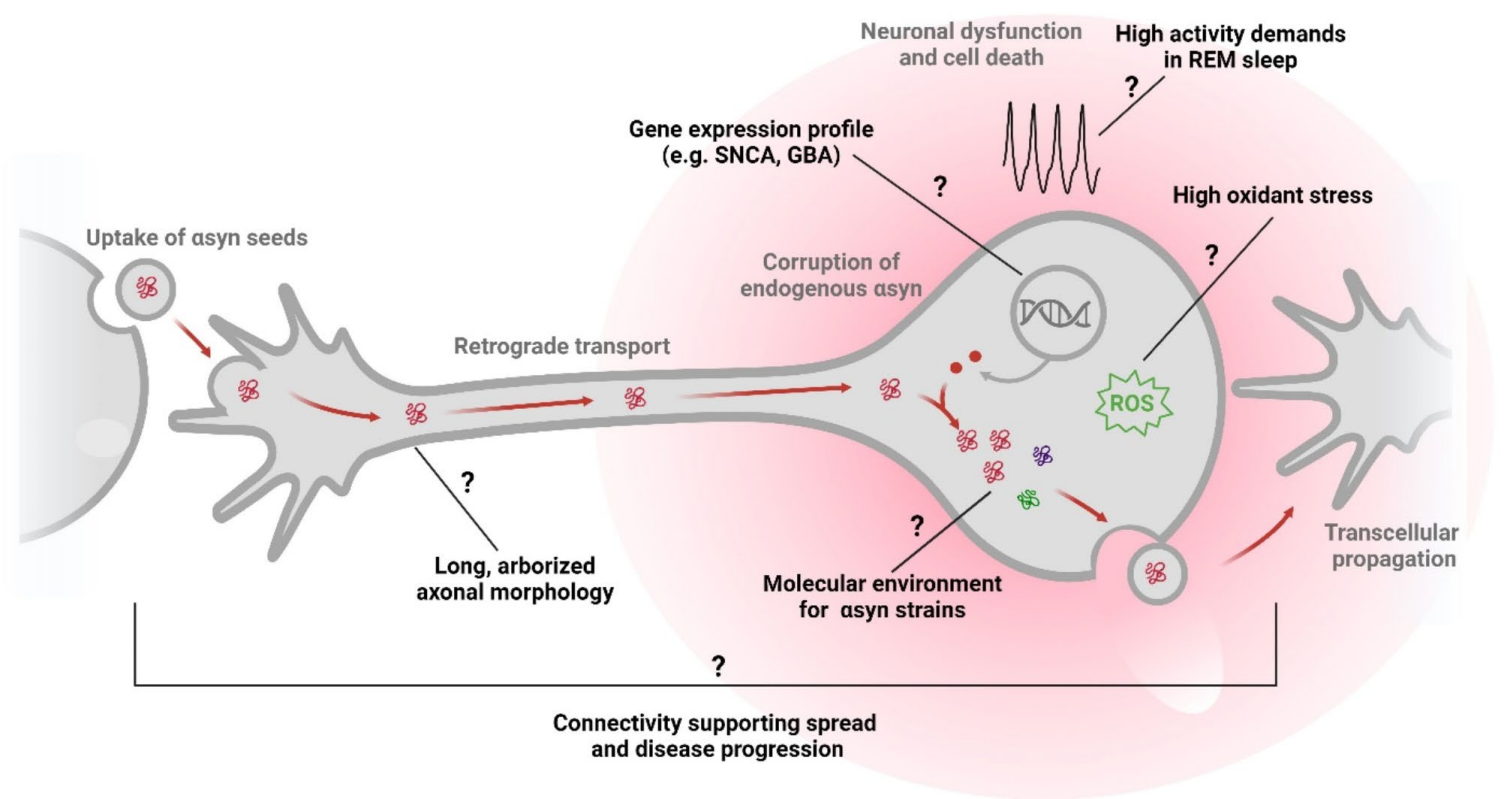
Factors speculated to contribute to the selective vulnerability of REM-atonia regulating neurons in RBD. Clinical progression from prodromal (i.e. RBD) to clinically manifest stages of synucleinopathy is thought to be driven by the spread of αsyn pathology along neuronal connections. The afferent and efferent connections of REM-atonia regulating brainstem populations such as the SLD and vM are largely consistent with caudal-rostral spread of pathology, which would give rise to RBD before Parkinson’s disease. The propagation of αsyn pathology can be further modulated by various cell-autonomous factors, including the cell milieu, axonal projections, gene expression, neuronal activity, and different αsyn conformational strains. Future investigations into how each of these factors modulates pathology specifically in the context of SLD and vM neurons would enrich our understanding of RBD mechanisms. Figure created with BioRender.com

One question that remains unaddressed is why RBD circuits are initially targeted by neurodegeneration. Examining the similarities between these circuits and other selectively vulnerable cell populations may provide insights into RBD pathogenesis. Several factors may contribute to the vulnerability of REM atonia-regulating regions to pathogenic α-syn (Fig. 2). Cell-autonomous factors, such as the firing properties of SLD and vM neurons [94, 95], their axonal projections [69, 70, 96, 97], and gene/protein expression levels could predispose these cells to pathology, as described for several nuclei with high pathologic burden in PD [98]. On the other hand, tract tracing [69, 70, 97, 99] and α-syn seeding experiments [93, 100] support the capacity of brainstem REM atonia circuits to transmit pathology along the caudal-rostral neuraxis, highlighting the role of connectivity in disease progression [101]. Additionally, the common occurrence of RBD among three distinct synucleinopathies, along with the existence of distinct α-syn conformational strains in each synucleinopathy [102–104], suggests a unique cellular milieu of SLD and vM neurons that supports the pathogenicity of different strains. Ultimately, we speculate that these factors likely intersect at the level of REM atonia-regulating circuits, driving their degeneration and resultant RBD symptoms. Further research is needed to assess each factor in the context of SLD and vM neurons and their contribution to RBD pathogenesis.

In summary, clinical and basic science evidence suggest that ɑ-syn pathology begins in the lower brainstem where REM atonia circuits are located, then propagates rostrally to brain regions (e.g., substantia nigra, limbic system, cortex) classically associated with PD and DLB. This observation is not only consistent with neuropathological evidence but is also in line with the early onset of RBD in the disease course of synucleinopathies. Therefore, the current thinking in the field is that iRBD is *is* one of the earliest detectable symptoms of synucleinopathies.

### Genetics and epigenetics in RBD

Compared to PD, much less is known about the genetic basis of RBD, yet in recent years our understanding of the genetic factors underlying RBD has considerably improved. Overall, there is only a partial overlap between the genetics of RBD, PD and DLB, suggesting that individuals with RBD, or more specifically those with iRBD, may represent a specific subpopulation within PD and DLB.

Thus far, the largest genetic study on RBD was a genome-wide association study (GWAS) that included about 1,000 patients with v-PSG-confirmed iRBD, about 1,700 PD patients with probable RBD and more than 140,000 controls [105]. This GWAS revealed several interesting observations. First, all five loci identified in the GWAS seem to revolve around the *GBA1* pathway. These loci include *GBA1* itself, *SNCA* which encodes α-syn and interacts with *GBA1* [106], *TMEM175* which regulates lysosomal activity of the enzyme encoded by *GBA1* – glucocerebrosidase [107], *SCARB2* which encodes the transporter of glucocerebrosidase from the endoplasmic reticulum to the lysosome [108], and the *INPP5F* locus which includes *BAG3*, which may be involved in folding of glucocerebrosidase [109]. It is therefore clear that *GBA1* has an important role in RBD, as was suggested in an earlier multi-center study [110]. Another study that genetically links *GBA1* to RBD showed that very rare loss-of-function mutations in *PSAP*, the gene that encodes saposin c (the co-activator of glucocerebrosidase) may occur together with *GBA1* mutations in RBD [111].

Other interesting findings in this GWAS were associations that are not observed, despite having sufficient power to detect them. For example, while the *MAPT* and *LRRK2* loci are among the top associations in PD GWAS [112], in the RBD GWAS they were not detected at all. This is consistent with previous reports suggesting lack of association between these two genes and RBD [113–115]. Similarly, the RBD GWAS did not find an association with *APOE*, which is the strongest risk factor identified in DLB GWAS [116]. These lack of associations exemplify the partial overlap between RBD and PD or DLB. Furthermore, in two of the loci that are shared between RBD, PD and DLB, there are different effects observed. In the *SNCA* region, different variants affect the risk for RBD and PD, as previously reported [117]. A similar phenomenon is seen in the *SCARB2* locus, where the top associations are different between RBD and PD. This might be explained by different effects of these variants on expression in different brain regions, some of which are more relevant for PD (e.g. substantia nigra), while others might be more specific for the subpopulation of patients with RBD [105].

There are no good studies on familial RBD forms as there are in PD, and screening of known familial PD and atypical parkinsonism genes in sporadic RBD, such as *PRKN*, *PINK1*, *LRRK2*, *VPS35* and others, found no evidence for the involvement of any of the 10 tested genes in RBD [114]. However, testing for the effect of rare variants in genes that are known to be involved in sporadic PD that were identified through the most recent PD GWAS [112], suggested that *BST1* and *LAMP3* may also be involved in RBD [118]. While *LAMP3* is another lysosomal related gene, which strengthen the notion of dysfunction of this organelle in RBD, *BST1* was the first genetic marker that may associate RBD to the immune system. More recently, fine mapping of the *HLA* genomic region suggested that several *HLA* types may also be associated with RBD [119]. However, these findings need to be replicated in additional studies.

Several recent studies have demonstrated that epigenetics may have an effect or serve as a marker for RBD progression. Epigenetics allow approximate determination of biological aging. Using this approach, it was shown that faster epigenetic aging may be associated with earlier age at onset of iRBD and faster phenoconversion [120]. Overall, iRBD patients seem to have accelerated aging based on epigenetic markers [121]. More specific findings suggested that hypomethylation of the *SNCA* locus maybe associated with iRBD and its phenoconversion to PD [122, 123].

One of the major caveats in genetic and epigenetic studies of iRBD is that they require a large number of participants. Once home-based diagnosis of iRBD will be possible, identifying and recruiting iRBD patients will become easier and facilitate much larger studies.

### What can we learn from neuroimaging in RBD?

Presynaptic dopaminergic imaging such as [^123^I]FP-CIT-SPECT, commonly named dopamine transporter (DaT) SPECT, is the most established neuroimaging biomarker in iRBD and identifies patients at high risk of short-term phenoconversion [14, 124, 125]. However, some methodological issues should be discussed. A dichotomous categorization of DaT-SPECT in normal/abnormal is an oversimplification, especially since a clear abnormality cut-off has not been defined yet. Of note, phenoconversion risk in iRBD patients widely changes by applying different cut-offs [126]. DaT-SPECT semi-quantification [124, 125] provides instead continuous metrics reflecting nigro-striatal dopaminergic function of different basal ganglia structures (such as putamen and caudate), as well as derived parameters (such as putamen/caudate ratios and asymmetries). ThusDaT-SPECT should be considered a stage biomarker, with degrees of abnormality depending on the disease stage (early to advanced), and with phenotype-related patterns of dopaminergic impairment. Following these concepts, a recent multicenter study showed that using specific cut-offs the most affected putamen best identified iRBD who later developed overt parkinsonism or dementia, while the most affected putamen/most affected caudate ratio identified patients who developed a parkinsonism-first instead of a dementia-first phenotype [125]. Thus, DaT-SPECT can be considered as a stratification biomarker in alpha-synucleinopathy. Notably, DaT-SPECT demonstrated progressive loss of nigro-striatal dopaminergic function in iRBD patients [127] and showed promising results as an explorative endpoint in a proof-of-concept neuroprotection study in iRBD patients [128]. Therefore, DaT-SPECT may also be considered as a neurodegeneration progression biomarker, at least for later stages of degeneration.

Brain MRI has been used to unveil structural and functional abnormalities, and their progressive development, in iRBD patients [129]. Iron accumulation [130–132]and neuromelanin loss [133–135] in the substantia nigra was found in iRBD patients. In a longitudinal study of RBD, striatal dopaminergic denervation occurred first followed by abnormal iron metabolism and finally neuromelanin changes in the substantia nigra pars compacta [132]. Recent studies highlighted cortical other than subcortical abnormalities in iRBD patients [136, 137], with brain atrophy correlating with both motor and cognitive impairment [136], suggesting the presence of a widespread neurodegeneration process already involving cortical brain areas since prodromal alpha-synucleinopathy stages.

The early involvement of the brain cortex in iRBD was also suggested by several [^18^F]FDG-PET studies. Indeed, iRBD patients already express the brain glucose metabolism pattern of overt PD [138, 139], and more severe brain glucose metabolism abnormalities were associated with an increased risk of short-term phenoconversion [139, 140]. Interestingly, patients’ expression of such patterns changes from prodromal to overt alpha-synucleinopathy stages [141, 142], suggesting that brain [^18^F]FDG-PET may be candidate neurodegeneration progression biomarker.

### Home detection and digital monitoring of RBD

The current diagnostic procedure for RBD requires video-polysomnography (vPSG) to demonstrate REM sleep without atonia (RSWA) [8], however vPSG analysis and interpretation requires specific training, and it is costly and not widely accessible. RBD questionnaires consist of a single item or a set of questions screening for abnormal movements and/or vocalizations related to dream content. However, patients are often unaware of their symptoms, and they have a low positive predictive value in the general population due to the relatively low prevalence of iRBD compared to mimics (e.g., obstructive sleep apnea, periodic limb movement disorder, sleep-related hypermotor epilepsy, other parasomnias causing abnormal behaviors) [10]. These limitations have provided the rationale for developing home-based solutions for diagnosing RBD. Broadly, three types of devices have been used to date.

#### Home vPSG systems

Seger et al. used a home vPSG system to evaluate RSWA in 124 community participants with a high pretest probability of RBD [143]. Although a portable one, the device used required labor-intensive manual scoring, and employed the same number of electrodes and sensors as conventional vPSG. A machine-learning model for diagnosing RBD based on 3D video analysis of REM sleep movements in a sleep laboratory has been developed [144, 145]. This approach has not yet been tested in the home environment where it would require automated detection of REM sleep or movement analysis throughout the night.

#### Head bands

One head band device was tested in 19 patients with iRBD. The device inputs signals from 3 frontopolar sensors (electroencephalogram and electrooculogram), one frontal electromyographic sensor, photoplethysmography (pulse), head movements and snoring [146]. Although potentially more acceptable than full vPSG, its auto-staging algorithm needs to be further validated [147].

#### Wrist-worn accelerometers (known as “actigraphs”)

Several studies have used wrist actigraphy to detect abnormal movements during sleep [148–151] and disruption of 24 h rest-activity rhythms (RAR) [148, 152–154]. Salient actigraphy features in RBD include increased total activity counts during sleep, clusters of movements coinciding with REM sleep cycles [151], and a relative reduction of diurnal activity counts. Used alone, single features of actigraphy do not have sufficient discriminative value in RBD, however their combination using machine learning is a promising approach for home diagnosis of RBD [148]. Advantages of a wrist wearable include high acceptability, low cost, and the possibility of recording multiple days/nights to raise accuracy. To date, three studies in distinct datasets of patients with iRBD [148, 155] or RBD secondary to PD [156] and controls with and without other sleep disorders have shown sensitivities of 79–95% and specificities of 92–96%. Current limitations of actigraphy include potential reliance on sleep diaries and the uncertainty around the transferability of existing algorithms to other devices and datasets.

What should digital approaches seek to measure for RBD progression? Multicentre studies demonstrate motor symptoms and signs show greater progression over time than non-motor features, with MDS-UPDRS-III then Purdue Pegboard, MDS-UPDRS II and Timed Up and Go scores showing greatest change [14, 157]. In RBD who later developed PD, voice changes and hypomimia appeared 9 years prior to diagnosis, followed by finger tapping deficits, mobility deficits, rigidity and limb bradykinesia [24]. Cognitive variables showed modest progression with higher variability, decline beginning 10 years prior to dementia conversion [26, 157]. Here, we summarise digital monitoring of motor and cognitive progression in RBD, a rapidly evolving space.

#### Wearables- body worn devices

Approaches to monitor gait in RBD found micro changes (reduced gait velocity, variability and rhythm) in home gait measured over 7 days using lower back tri-axial accelerometry [158]. Subtle in-clinic gait deficits during fast-paced and dual-task walking using multiple sensors are also present [159–162].

#### Mobile technologies and applications

Finger-tapping deficits with impaired spontaneous rhythm production or amplitude decrement using a tablet/3D motion capture system are present in RBD compared to controls [163, 164]. Motor deficits including tremor, tapping speed, gait and voice changes were demonstrated in RBD and PD compared to controls using smartphones [165]. Smartphone capture of spontaneous speech abnormalities in RBD allowed separation from controls [166, 167], while spoken language alterations predicted phenoconversion [168].

#### Home-based computers and sensing systems

A brief online cognitive battery via home PC in 50 control, 59 PD and 54 RBD participants showed greater sensitivity to memory, language, attention and executive function deficits than supervised neuropsychological scales [169]. Metacognitive accuracy in RBD and PD aligned with controls, indicating subjects are generally aware of their cognitive status [170]. Home sensing systems have not been evaluated in RBD, although a recent study found the approach broadly acceptable for PD participants [171].

Home detection and digital monitoring of RBD are however still being developed and currently vPSG cannot be replaced as diagnostic instrument, although screening possibilities are evolving and likely to further develop in the near future.

### Biofluid and tissue markers of neurodegeneration

During the last few years research focused on early detection of α-syn alterations in biofluids and tissues (Table 1). Among biofluids, cerebrospinal fluid (CSF) is by far the one expected to bring the most relevant results, even if slightly invasive. The diagnostic performance of α-syn-seed amplification assays (α-syn-SAAs) for patients with RBD showed sensitivity rates of 0.64 (95% CI, 0.50–0.77) [172], 0.80 (95% CI, 0.58–0.92) [173] and 1.00 (95% CI, 0.82–1.00) [174]. A recently published cross-sectional study evaluating the PPMI at-risk groups indicated a sensitivity of SAA assays in 86% of subjects with iRBD/hyposmia and 8% of mutation carriers [175]. Unfortunately, only a few studies included longitudinal assessment. Iranzo and co-authors found a diagnostic accuracy of 90% in predicting the phenoconversion of iRBD, considering a follow-up interval of up to ten years [173].

**Table 1:** Biofluids and tissues markers in iRBD

Technique	Biological Sample	Cost Invasiveness Sensitivity/specificity	Prodromal PD cross-sectional	Prodromal PD longitudinal
Total αSyn	CSF	+/- + NA	-	-
oligomeric αSyn (ELISA)	CSF Blood	+/- + NA +/- + NA	++ NE	- NE
RT-QuIC αSyn	CSF Saliva GI Biopsy Skin Olfactory Mucosa	+ + +++/+++ +/- - NA + ++ -/- + +/- +++/+++ + +/- +/++	++ - ++ ++ +	+ - - ++ -
NfL	CSF Blood	NA - - ++/-	- ++	- ++

NA: non available

NE: non evidence

Other tissues may also serve for α-syn detection. Nasal brushing for detecting α-syn aggregates by α-syn SAA showed positivity in 44% of the patients with iRBD (particularly those with coexistent hyposmia), 46% with manifested PD and 10% of controls without RBD [176]. Immunohistochemistry for phosphorylated α-syn (p-α-syn) was first reported in colonic tissue, although with a very low positivity rate (24%) [177]. Higher sensitivity (89%) was then obtained with submandibular gland biopsy. Still, adequate biopsy material can be obtained in less than half of the patients [178], while minor salivary glands biopsy obtained adequate tissue in all cases, although showing lower sensitivity (50%) [179].

Lately, detection of p-α-syn with *fluorescence immunohistochemistry* analysis of skin biopsies emerged as a promising and less invasive technique [180, 181] with a sensitivity of up to 86.7% [182] and a high specificity (up to 100%).

When comparing detection of α-syn by means of *fluorescence immunohistochemistry* and α-syn SAAs in skin and CSF, the first seems to have greater diagnostic accuracy [183]. However, Iranzo et al., by comparing the results of αSyn SAA in the skin and CSF, performing both sampling on the same morning, in 91 patients with iRBD and 41 controls, found similar high sensitivity (> 75%), specificity (> 97%) [184],, and a near perfect concordance between skin and CSF results. A large study in 148 iRBD patients showed CSF synuclein positivity in 75%, decreased Aβ_42_/Aβ_40_ ratio in 22%, increased phosphorylated tau in 16%, increased total tau in 15%, and elevated neurofilaments (NfL) in 15%. In the CSF, only synuclein positivity was a marker of short-term overall phenoconversion, while elevated p-tau/Aβ42 was predictive of DLB [185]. 

Biomarkers have been researched also in the blood which of course carries less invasiveness for the patients. Two longitudinal studies using the SIMOA technique indicated NfL as a promising biomarker in reflecting disease severity of iRBD and predicting disease progression and phenoconversion [186, 187]. Among other blood biomarkers, also increased α-synuclein measured by immunocapture in neuronally derived extracellular vesicles has been proposed for the stratification of patients at higher risk of phenoconversion [188]. Recently, by applying a machine-learning model to a targeted multiplexed mass spectrometry assay for blood samples, authors could isolate a specific blood panel of molecular events for identifying at-risk participants [189].

Further longitudinal studies are needed to understand sensitivity and specificity of biofluids and tissues biomarkers and also their potential role as prognostic biomarkers. Future engagement in improving these techniques with quantitative measurements for prognostic aims is warranted.

### The role of RBD in the new staging systems of Parkinson’s disease

Recent biomarkers advances, including detection of misfolded α-synuclein aggregates through seed amplification assay, led to proposal of two similar but conceptually different approaches to improve integration of biological information in the definition and classification of PD or neuronal α-synuclein diseases. This is relevant also for iRBD, as this prodromal stage is included in the proposed staging systems.

The SynNeurGe classification [190] is a biological classification of PD aiming at advancing basic science and clinical knowledge eventually leading to precision medicine for disease-modifying treatments. This classification uses three components: pathological α-synuclein (Syn) in tissues or CSF; neurodegeneration (Neur) as defined by dopaminergic deficit demonstrated by neuroimaging; and pathogenetic gene variants (Ge) causing or strongly predisposing to PD. A clinical component is also included, in order to define whether a given clinical syndrome can be attributed to biologically-diagnosed PD. Either a single high-specificity clinical feature or multiple lower-specificity clinical features can be used. RBD is considered possibly related to PD is not PSG-confirmed, and probably related to PD if PSG-confirmed. No distinction between prodromal and defined disease stages is present. Of note, none of these components is required, to account for the biological heterogeneity of Parkinson’s disease.

The neuronal α-synuclein disease integrated staging system (NSD-ISS) [191] proposes a new definition of PD and DLB under the common concept of neuronal α-synuclein disease, based on the in vivo detection of pathological neuronal α-synuclein, regardless of any specific clinical features. Accordingly, stage 0 is defined by the presence of pathogenic variants in the SNCA gene; stage 1 requires presence of pathological neuronal α-synuclein, alone (stage 1 A), or with dopaminergic neuronal dysfunction (stage 1B). Subtle clinical manifestations without functional impairment (including iRBD) mark stage 2, with sub-classification based on dopaminergic neuronal dysfunction. Stages 3 to 6 present stage-specific increases in functional impairment.

As knowledge and biomarkers develop, a shift towards a biologically driven diagnosis is desirable. However, several aspects need to be carefully considered. These include reliability and availability of proposed biomarkers, and most relevantly their clinical significance. Thus far, no data are available to inform if subjects with pathological α-synuclein will develop clinical symptoms, when symptoms would develop, and if those would be predominantly motor or cognitive. Adopting such classification and staging methods without a clear answer to these issues raises ethical issues, which are particularly relevant for patients with isolated RBD. iRBD is already recognized as a prodromal stage of α-synucleinopathy, as the vast majority of these patients will develop a clinically manifest α-synucleinopathy over time [14]. However, those patients may be excluded from clinical trials on disease-modifying treatments if they present no pathologic α-synuclein. Moreover, those going to phenoconvert into multiple system atrophy are not represented in the proposed classification and staging methods. The peculiarity of iRBD as prodromal synucleinopathy should not get lost in such proposals.

These newly proposed staging systems of PD may be still be biased by oversimplification, focusing only on one abnormal protein (synuclein), one neurotransmitter (dopamine), one cell (neuron) and neglecting molecular (e.g., lysosomal, mitochondrial) and neuropathological (comorbid proteinopathies) data. A post-mortem examination of 20 brains of patients with PSG-proven RBD, isolated or who evolved to PD or DLB before death, showed that all had a postmortem synucleinopathy but all also had abundant synuclein deposits in the astrocytes, and exhibited co-pathologies [192], including Alzheimer’s disease pathology (i.e. amyloid β plaques and neurofibrillary tangles), ageing-related tau astrogliopathy, cerebral amyloid angiopathy, argyrophilic grain disease, limbic-predominant age-related TDP-43 encephalopathy, and early changes indicative of progressive supranuclear palsy). These aspects need to be taken into account in future staging systems.

### Towards clinical trials in iRBD

There may be no group more ideally placed for synucleinopathy neuroprotective trials than iRBD patients, because:

1. They already have a neurodegenerative disease — more than 80% would qualify for biological PD in the SyNeurGe classification, with a slightly lower proportion in the NSD classification [190, 191].
2. They are not receiving symptomatic therapy– The need to start symptomatic therapy dramatically impairs the successful conduct of neuroprotective trials in PD. For example, 65% of de novo PD patients in the PPMI study started symptomatic therapy within the first year [193]. Removal of this confound allows shorter trials in patients with iRBD, i.e. 2–3 years.
3. They are at earlier stages, implying room to intervene. Almost all dopaminergic SNpc neurons are already lost within a few years of PD diagnosis, while by contrast, 60–70% of iRBD patients still have normal dopamine transporter scans [124].

### Selection of the intervention

Although many agents currently in development for PD and DLB may be useful for iRBD, there are some special considerations. First, an agent should preferably be useful against both forms of synucleinopathy, at least unless careful stratification is used. It must also target mechanisms of disease that are present early in the disease course, and those that are evident in PD/DLB patients with iRBD. Finally, as iRBD participants still generally feel relatively well and so may be less tolerant of invasive interventions, the agent should be relatively safe and easy to administer.

This suggests several options. Synuclein is the obvious primary target, and options include including passive immunotherapy, active immunotherapy, aggregation inhibitors, antisense oligonucleotides, etc. Lysosomal dysfunction also seems to be a critical in iRBD pathophysiology, and agents targeting glucocerebrosidase A-related mechanisms are currently in trials in PD (e.g. GCAse activators and chaperones). Finally, neuroinflammation is prominent early in iRBD, and so agents targeting inflammation (e.g. inflammasome targets, already-approved immunotherapy agents) also hold promise.

### Patient inclusion

Accurate patient inclusion is essential to success. Some key considerations include:

1. Ensuring synucleinopathy is present. While most PSG-proven iRBD have underlying synucleinopathy, some may not. RBD can be triggered by antidepressant medications, or associated with brainstem lesions, autoantibody syndromes, etc [194]. A second positive test, such as synucleinopathy documentation in biofluids and tissues via highly specific assays, can help ensure the patient is ‘on target’ for intervention. However, follow-up data about false negative cases is still limited, large-scale operationalization is not yet available, and delays can complicate screening. Recent studies found strong correlations between olfactory loss and synuclein seeding assays [175]; suggesting that olfactory loss documentation may serve as sufficient secondary surrogate confirmation, pending longitudinal data on individuals with olfactory loss.

Video-polysomnographic confirmation of iRBD is the gold standard for diagnosis. However, it should be remembered that the goal of disease modifying trials is to detect early prodromal synucleinopathy, not iRBD per se. Given resource limitations, using a clear RBD history combined with strong ancillary evidence of degeneration (e.g., detection of synuclein in biofluids or tissues, dopaminergic imagings) might be considered to identify early-stage synucleinopathy. Additional lower-specificity tests for RBD itself (actigraphy or home-based PSG devices) may increase diagnostic accuracy further [148, 156], notwithstanding currently available literature supports higher accuracy of vPSG confirmed iRBD as early stage synucleinopathy.

2. The late-stage vs. generalizability trade-off. Selecting all RBD patients aids recruitment and ensures early intervention, but selecting more advanced patients may yield more observable decline, reducing sample size and study duration. Suggested stratification measures like mild motor/cognitive impairment or dopaminergic denervation can be considered, although this implies having < 50% of patients eligible and risk of treatment failure if these patients are already too close to phenoconversion at the time of inclusion.
3. Would you select IRBD patients with negative synuclein in the CSF (about 25%)?

## Outcome selection

Choosing the most appropriate primary outcome is critical. Primary options include:

1. Biomarkers: Earlier phase studies generally rely upon sensitive biomarkers, even with uncertain clinical application. However, in prodromal PD, there may not be sufficiently sensitive biomarkers; some studies suggest slight numerical advantages to presynaptic dopaminergic imaging [124], though sample size advantages are modest.
2. Phenoconversion: This is the most direct marker, although there can be challenges with standardization and higher sample size/duration requirements.
3. Clinical scales– Scales for parkinsonism and impaired cognition are broadly validated. A recent analysis of the International RBD Study Group concluded the most efficient clinical outcome was a clinically-meaningful decline in motor and/or cognitive scales, analyzed categorically as a time-to-event. Sample sizes using this measure ranged from 121 (50% effective agent, 3-year study) to 742 (30% effective agent, 2-year study) patients per group [157].
4. Regardless of design details, the compelling advantages of iRBD implies multiple avenues forward. The field is clearly ready to start neuroprotective trials.

## Data Availability

Not applicable.
